# Neoadjuvant chemotherapy induces an elevation of tumour apparent diffusion coefficient values in patients with ovarian cancer

**DOI:** 10.1186/s12885-023-10760-2

**Published:** 2023-04-01

**Authors:** Milja Reijonen, Erikka Holopainen, Otso Arponen, Mervi Könönen, Ritva Vanninen, Maarit Anttila, Hanna Sallinen, Irina Rinta-Kiikka, Auni Lindgren

**Affiliations:** 1 Department of Radiology, Tampere University Hospital, Tampere, Finland; 2 Faculty of Medicine and Health Technology, University of Tampere, Tampere, Finland; 3 Department of Radiology, Kuopio University Hospital, Kuopio, Finland; 4 Institute of Clinical Medicine, School of Medicine, Clinical Radiology, University of Eastern Finland, Kuopio, Finland; 5 Department of Clinical Neurophysiology, Kuopio University Hospital, Kuopio, Finland; 6 Department of Gynaecology and Obstetrics, Kuopio University Hospital, Kuopio, Finland; 7 Institute of Clinical Medicine, School of Medicine, Obstetrics and Gynaecology, University of Eastern Finland, Kuopio, Finland

**Keywords:** Ovarian cancer, Magnetic resonance imaging, Diffusion-weighted imaging, Apparent diffusion coefficient, ADC values, Region of interest, Neoadjuvant chemotherapy

## Abstract

**Objectives:**

Multiparametric magnetic resonance imaging (mMRI) is the modality of choice in the imaging of ovarian cancer (OC). We aimed to investigate the feasibility of different types of regions of interest (ROIs) in the measurement of apparent diffusion coefficient (ADC) values of diffusion-weighted imaging in OC patients treated with neoadjuvant chemotherapy (NACT).

**Methods:**

We retrospectively enrolled 23 consecutive patients with advanced OC who had undergone NACT and mMRI. Seventeen of them had been imaged before and after NACT. Two observers independently measured the ADC values in both ovaries and in the metastatic mass by drawing on a single slice of (1) freehand large ROIs (L-ROIs) covering the solid parts of the whole tumour and (2) three small round ROIs (S-ROIs). The side of the primary ovarian tumour was defined. We evaluated the interobserver reproducibility and statistical significance of the change in tumoural pre- and post-NACT ADC values. Each patient’s disease was defined as platinum-sensitive, semi-sensitive, or resistant. The patients were deemed either responders or non-responders.

**Results:**

The interobserver reproducibility of the L-ROI and S-ROI measurements ranged from good to excellent (ICC range: 0.71–0.99). The mean ADC values were significantly higher after NACT in the primary tumour (L-ROI *p*  < 0.001, S-ROIs *p* < 0.01), and the increase after NACT was associated with sensitivity to platinum-based chemotherapy. The changes in the ADC values of the omental mass were associated with a response to NACT.

**Conclusion:**

The mean ADC values of the primary tumour increased significantly after NACT in the OC patients, and the amount of increase in omental mass was associated with the response to platinum-based NACT. Our study indicates that quantitative analysis of ADC values with a single slice and a whole tumour ROI placement is a reproducible method that has a potential role in the evaluation of NACT response in patients with OC.

**Trial registration:**

Retrospectively registered (institutional permission code: 5302501; date of the permission: 31.7.2020).

## Background

Worldwide, ovarian cancer (OC) was the eighth most common cancer in women in 2020 [1]. The 5-year survival rate of OC patients varies between 29 and 92% according to the stage of the disease. OC is the most lethal gynaecological cancer [2]. Most (90%) OCs are of the epithelial subtype. Rapidly evolving, heterogeneous high-grade serous ovarian cancer (HGSOC) is the most common epithelial OC and one of the aggressive histological subtypes [2–6]. At the time of diagnosis, approximately two-thirds of epithelial OCs are Federation of Gynaecology and Obstetrics (FIGO) stage III or IV and present with signs of extra-ovarian dissemination, such as peritoneal and omental nodules, and high-volume ascites [4, 7].

The standard treatment of FIGO stage III–IV epithelial OC has traditionally been a combination of radical primary debulking surgery (PDS) with the aim of completely resecting all the macroscopic disease (R0), followed by platinum-based adjuvant chemotherapy [8, 9]. However, several trials focusing on patients with advanced OC (FIGO stages IIIC–IV) have demonstrated that those treated with neoadjuvant chemotherapy (NACT) have better surgical outcomes and a lower risk of postoperative morbidity [10–13]. Indeed, the National Comprehensive Cancer Network (NCCN) guidelines, the European Society of Medical Oncology (ESMO), and the European Society of Gynaecological Oncology (ESGO) recommend NACT with interval debulking surgery (IDS) for selected patients who are poor candidates for surgery (e.g., due to advanced age, comorbidities, or frailty) or who are unlikely to achieve optimal cytoreduction in primary debulking surgery due to the large spread of the disease based on clinical or radiological markers [10, 14, 15].

The shifting paradigm of NACT in OC challenges conventional imaging practices. The European Society of Urogenital Radiology (ESUR) recommends magnetic resonance imaging (MRI) as the modality of choice when imaging an indeterminate pelvic or adnexal mass, for example, when OC is suspected following a transvaginal ultrasound [16]. In addition to its role in helping with the differential diagnostics, preoperative MRI aids in the evaluation of the resectability of OC patients [17, 18]. Furthermore, MRI has been shown to have high accuracy in terms of revealing the extent of peritoneal carcinomatosis and other difficult-to-resect sites or anatomic abnormalities, which may lead to suboptimal surgical outcomes [18]. Several MRI techniques have also been used successfully to assess the stage of the disease and the response to chemotherapy in OC. Multiparametric MRI (mMRI) techniques have, additionally, proved to be valuable when assessing the histological severity and prognosis of OC [19, 20]. Diffusion-weighted imaging (DWI) and dynamic contrast-enhanced (DCE) sequences are known to improve the ability to distinguish between benign and malignant tumours when combined with the routine anatomic T1- and T2-weighted (T1W and T2W) sequences [16, 19, 20].

DWI is a structural MRI technique that provides insight into a tumour’s microscopic tissue architecture, such as tumour cellularity, fluid viscosity, and cell membrane integrity, by detecting the level of water diffusion, which can be quantified with apparent diffusion coefficient (ADC) values. Low ADC values indicates high cellularity and an increase in water restriction within the tumours, both of which might change in response to chemotherapy [21]. Multiparametric MRI allows a more beneficial phenotypic characterization of tumours and might improve the determination of NACT response assessment and prognostic evaluation [22]. Indeed, when imaged before to any treatment (PDS or NACT), reduced ADC values in OC patients have been associated with lower 3-year survival rate [19]. Winfield et al. [23] showed that a greater increase in ADC values between pre- and post-NACT scans was a predictive marker of NACT response in OC patients. However, the analysis of ADC values has not been standardized for patients with OC. In the literature, the regions of interest (ROI) used in ADC measurements vary from single to multiple slice measurements, and the ROI sampling method (e.g., round, square, or freehand) has been proposed. The ROIs have also been drawn with interactive computer-assisted segmentation by indicating the target lesion [19, 23–27].

We hypothesized that the ADC values of both the primary tumour in the ovaries and the omental metastasis would increase significantly between the pre- and post-NACT scans. The rationale for this retrospective study was to compare the performance and evaluate the reproducibility of two ROI sampling methods (small round ROIs vs. a large freehand ROI) in the assessment of the change in ADC values in patients with OC receiving NACT.

## Materials and methods

### Study protocol and patients

We retrospectively reviewed consecutive epithelial OC patients who had been treated with NACT at Kuopio University Hospital, Finland, between 2011 and 2020. We included patients with histopathologically confirmed primary OCs (tissue material collected through an ultrasound-guided needle biopsy or a surgical procedure before proceeding to NACT) who were treated with platinum-based NACT prior to the IDS attempt, and who had been imaged with mMRI after NACT. The permit for the use of patient register data for research purposes was issued by the Chief Medical Officer of the hospital district (study number: 5302501). The need for written informed consent from the patients was waived in accordance with local laws and regulations on the basis of the study’s retrospective nature.

We enrolled 23 patients who had completed three to eight cycles of platinum-based NACT. Seventeen (74%) of those patients had been imaged with mMRI at the time of diagnosis (pre-NACT) and prior to surgery (post-NACT) and thus were available for the paired analysis of the change in the ADC values in response to NACT and for the reproducibility analyses. The other six (26%) patients had been imaged only with computed tomography (CT) at the time of diagnosis but were imaged with mMRI prior to surgery. They were thus included in the statistical assessment of the reproducibility of the two ROI sampling methods. Only one mid-treatment MRI was performed, and we therefore did not analyse it as a part of this study.

### Imaging protocol and image analysis

**Fig. 1:**
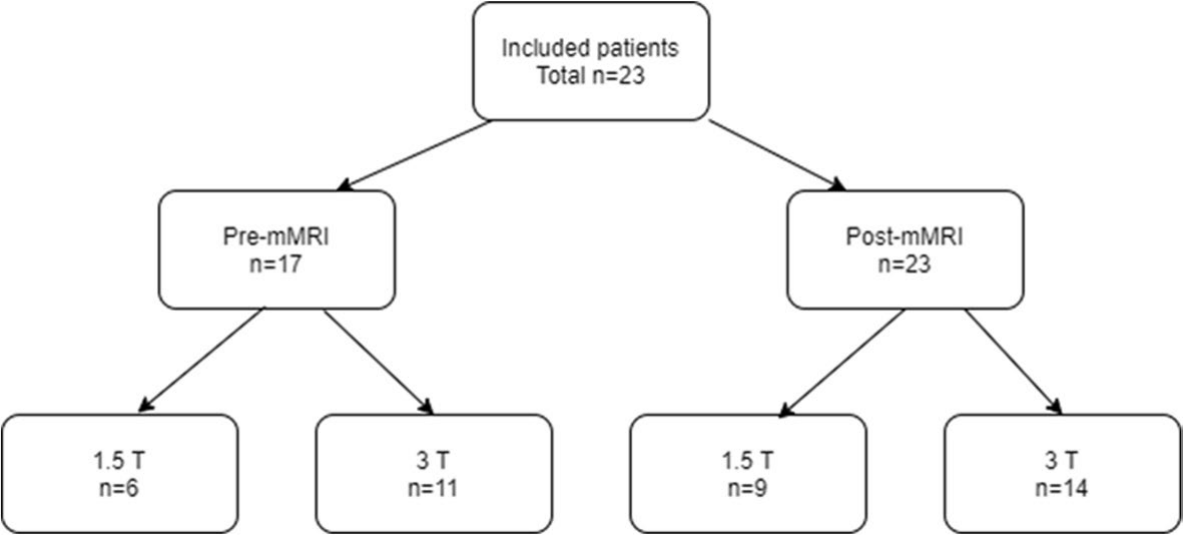
The distribution of the imaging protocols used in the study

Nine (39.1%) patients had been imaged with a 1.5 T MRI scanner (Body array coil, Siemens MAGNETOM Avanto/Avanto Fit/Aera, Siemens Healthcare GmbH, Erlangen, Germany) and 14 (60.9%) patients with a 3.0 T MRI scanner (Sense-XL-Torso coil, Philips Achieva 3.0 T X, Philips N.V., Eindhoven, The Netherlands). Those patients who had been imaged before and after the NACT treatments had consistently undergone imaging with a scanner of equivalent field strength (i.e., no crossover from 1.5 T to 3.0 T or vice versa) from the same MRI manufacturer. All the patients had undergone axial DWI protocols in the pelvic area. Fifteen (88.2%) patients in the pre-NACT imaging and 22 (95.7%) patients in the post-NACT imaging had also undergone DWI in the upper abdominal area (Fig. 1)*.* Monoexponentially derived ADC maps had been automatically generated with low *b*-value images (0 s/mm^2^ or 50 s/mm^2^), and high *b*-value images (600 s/mm^2^, 800 s/mm^2^ or 1000 s/mm^2^) were available for every patient. The detailed imaging protocol in DWI sequences is described in Table 1.

**Table 1:** An overview of the imaging acquisition protocols of the DWI sequences

Scanner	Coil	Parameters (range)	Pre-treatment		Post-treatment	
			**Lower abdomen**	**Upper abdomen**	**Lower abdomen**	**Upper abdomen**
**Philips Achieva 3.0 T**	SENSE-XL-Torso	**Number of patients (*n*)**	11	10	14	13
		**Slice thickness (mm)**	5.00	5.00	5.00	5.00
		**Repetition time (ms)**	1497–3912	480–3819	1793–4662	480–3759
		**Echo time (ms)**	48.00–50.60	48.00–51.80	47.90–50.60	48.00–50.80
		***Number of acquisitions (ACQ)***	4.00	4.00	4.00	4.00
		***Field-of-view (FoV, mm)***	350–450	327–450	360–453	360–453
		**Slices**	120–144	138–144	138–156	126–144
		**b-values (s/mm**^**2**^**)**	0, 300, 600, 800 (DWIBS^a^)	0, 300, 600, 800 (DWIBS^a^)	0, 300, 600, 800 (DWIBS^a^)	0, 300, 600, 800 (DWIBS^a^)
		**Matrix dimensions (range)**	(192×192 – 240×240)	(192×192 – 288×288)	(192×192 – 240×240)	(224×224 – 288×288)
**Siemens Avanto 1.5 T**	Body array	**Number of patients (*n*)**	3	1	6	6
		**Slice thickness (mm)**	6.00	6.00	6.00	6.00
		**Repetition time (ms)**	1700–4900	3939	1700–1820	1700–1820
		**Echo time (ms)**	72.00	76.00	62.00	62.00
		***Number of acquisitions (ACQ)***	2–3	2.00	2.00	2.00–5.00
		***Field-of-view (FoV, mm)***	284×379	301×400	328×420	328×420
		**Slices**	90–120	120	90–105	108–120
		**b-values (s/mm**^**2**^**)**	50, 200, 400, 800	50, 400, 1000	0/50, 400, 800	0/50, 400, 800
		**Matrix dimensions (range)**	144 × 192	104 × 138	300 × 384	300 × 384
**Siemens Avanto Fit 1.5 T**	Body array	**Number of patients (*n*)**	2	1	3	3
		**Slice thickness (mm)**	6.00	6.00	6.00	6.00
		**Repetition time (ms)**	1700–4900	3939	1700–4900	3732–5543
		**Echo time (ms)**	72.00	76.00	72.00	72.00–76.00
		***Number of acquisitions (ACQ)***	2–3	2	2–3	2
		***Field-of-view (FoV, mm)***	284×379	301×400	299×399—284×379	284×379—301×400
		**Slices**	90–120	120	90–120	90–120
		**b-values (s/mm**^**2**^**)**	50, 200, 400, 800^b^	50, 400, 1000	50, 200, 400, 800^b^	50, 400, 800/1000
		**Matrix dimensions (range)**	144×192	104×138	144×192	(104×138 – 144×192)
**Siemens Aera 1.5 T**	Body array	**Number of patients (*n*)**	1	1	0	0
		**Slice thickness (mm)**	6.00	6.00	n/a	n/a
		**Repetition time (ms)**	1700	1700	n/a	n/a
		**Echo time (ms)**	60	60	n/a	n/a
		***Number of acquisitions (ACQ)***	2	2	n/a	n/a
		***Field-of-view (FoV, mm)***	328×420	328×420	n/a	n/a
		**Slices**	90	120	n/a	n/a
		**b-values (s/mm**^**2**^**)**	0, 400, 800	50, 400, 800	n/a	n/a
		**Matrix dimensions (range)**	300×384	300×384	n/a	n/a
**Spatial resolution = FoV / Matrix dimensions**						

^a^Diffusion-weighted imaging with background body signal suppression

^b^a *b*-value 200 and/or 400 was used, *n/a* not applicable

The radiological responses to NACT had initially been evaluated by experienced radiologists based on changes in the sizes and signal intensities of the tumoural lesions. While taking into account all the performed MRI sequences using the localizer in Sectra PACS (IDS7, version 17.3.6; Sectra Imtec, Linköping, Sweden), the two observers (M.R and E.H, with 6 months and 2.5 years of experience in gynaecological MRI, respectively) independently measured the mean and minimum ADC values from the  MRI data using the ImageJ tool (version 1.8.0_112 for Windows 10, National Institutes of Health, Bethesda, MD, USA). The two observers performed the measurements blinded to each other’s ROIs and measurements, the histopathology of the tumour and the clinical course of each patient. In situations when one of the observers was in doubt about whether to include the ADC measurement (e.g., when the image quality was suboptimal), the cases were discussed and corrected, if necessary. The ADC values of both ovaries and omental masses were measured when possible.

We chose two 2-dimensional ROI sampling methods. The axial slices where the ovaries, the possible ovarian tumours, and the omental tumour masses were best visualized were chosen for the analyses. First, measurements were performed by drawing, freehand, a large ROI (L-ROI) that covered the whole solid tumour in each ovary and separately in the largest metastatic omental tumour mass (or carcinosis, when present). Cystic areas were avoided. The largest area was 4677 pixels in the ovaries and 6026 pixels in the omental mass. Second, when the tumoural area was large enough, one to three small, round ROIs (S-ROIs), with a set size of 5×5 pixels, were fitted inside the area first covered by the L-ROI on the same slice without targeting the lowest ADC areas. The ROIs were placed on the solid part of the residual ovary, while taking into account all the available MRI sequences when there were no significant areas of diffusion restriction left due to response to treatment in the post-NACT images. Muscle and fat reference values were obtained by drawing L-ROIs and one to three S-ROIs on axial slices depicting the right psoas muscle and subcutaneous fat. When measuring the muscle and fat values, if the same reference measuring point was not suitable for pre- and post-imaging due to image positioning, the reference value was not used in the statistical analysis (muscle measurements excluded: *n* = 4).

The change of ADC values during the NACT treatment (ΔADC) was defined as the change between the pre- and post-treatment values for tumours, ovaries, omental masses, and muscle and fat reference values. The percentual change in the ΔADC (ΔADC%) was calculated as (ADC_post-NACT_ – ADC_pre-NACT_) × 100 / ADC_pre-NACT_, where ADC_pre-NACT_ and ADC_post-NACT_ denote the mean ADC values in pre-treatment and post-treatment measurements, respectively.

### Clinical parameters

The clinical data, including the information regarding the final histopathology of the tumour, was retrospectively collected from the patients’ medical reports. The cancer treatment plan (i.e., either primary radical surgery or starting with NACT) was determined in a multi-disciplinary team (MDT) meeting. The patients were deemed either responders or non-responders by combining the results of the radiological response evaluation (e.g.. whetherthere were changes in the tumour size) and the approximate amount of visible tumour or carcinosis in cytoreductive surgery. The patients’ diseases were defined as platinum-sensitive (over 6 months without chemotherapy before recurrence) or resistant (continuous chemotherapy or an interval of less than 6 months until recurrence). A macroscopic residual tumour in IDS was recorded as *presence of residual tumour in surgery*, which included residual tumours of both ≤ 1 cm and > 1 cm, or no visible residual disease (0 mm). Cancer recurrence status was given as one of the following three categories: no recurrence, recurrent disease, or progressive disease, when the disease progrediated directly after adjuvant chemotherapy.

For statistical purposes, the side with the primary ovarian tumour was determined later, primarily on the basis of the pathology report that had been drawn up on debulking surgery. In the case of a bilateral tumour (*n* = 6, 26%), the larger tumour with radiologically more malignant features was chosen. The number of NACT treatments was dichotomized as standard or extended in length (three to four cycles or more than four cycles, respectively).

Progression-free survival (PFS) was defined as the interval between the date of the IDS (or when the tumour was still inoperable based on the post-MRI evaluation (*n* = 2, 8.7%), the end date of chemotherapy) and the date of diagnosed disease recurrence or progression (or when no disease recurrence or progression occurred, the end date of follow-up). Overall survival (OS) was defined as the period between the date of IDS or the end date of chemotherapy (*n* = 2, 8.7%) and the end date of follow-up or death. Because of the wide follow-up range, we decided to indicate the PFS and OS in percentual numbers of cancer recurrence or deaths in 1-, 2- and 3-year timepoints.

### Statistical analyses

We used the IBM SPSS Statistics for Windows 10 platform (version 26.0, 2019, IBM Corp, Armonk, NY, USA) for the statistical analyses. A confidence interval of 95% was determined, and a *p* value of ≤ 0.05 was selected to indicate statistical significance. Continuous variables are presented as means ± *SDs*, or as median and range values. Nominal variables are presented as crude numbers and percentages.

We calculated the intraclass correlation coefficients (ICCs) to determine the interobserver reproducibility of the different ADC sampling methods. The ICC values were interpreted to indicate poor (ICC = 0.00–0.20), fair (ICC = 0.21–0.40), moderate (ICC = 0.41–0.60), good (ICC = 0.61–0.80), or excellent (ICC = 0.81–1.00) reproducibility [18, 22].

Only the measurements by Observer 1 were used for other statistical analyses. For L-ROI measurements, the mean ADC value was used (L-mean). If the tumour was too small for three separate S-ROI measurements, fewer than three S-ROIs were measured. In the analysis of S-ROI, we used the lowest mean (S-mean_low_), the lowest minimum (S-min_low_), the average mean (S-mean_av_), and the average minimum (S-min_av_) ADC values of the one to three measured S-ROIs, as there is no clear consensus in the literature on the optimal way to perform ADC measurements. For the comparison between the four S-ROI variables mentioned above, we used the Bonferroni corrected p-value of p_adjusted_ = 0.05 / 6 = 0.00833.

The Kolmogorov–Smirnov and Shapiro–Wilk normality tests were employed to assess the normality of the ADC measurements. The paired t-test was used to analyse the change between the pre- and post-NACT ADC values. The Wilcoxon-signed rank test was used for non-normally distributed variables to analyse the difference between the ADC values in the primary ovarian tumour and those in the omentum. The Kruskal–Wallis test was used to test the statistical significance of the relationship between ADC measurements and multicategory variables, namely cancer recurrence. The Mann–Whitney U test was employed to analyse the associations between the ADC values and clinical dichotomized parameters (platinum sensitivity and residual tumour status). The Spearman correlation test was chosen to investigate the statistical correlations between the clinical continuous variables (i.e. age, body mass index [BMI] and the cancer antigen 125 [CA-125]) and averaged ADC values.

We used the Cox regression analysis for progression-free and overall survival analyses with continuous ADC variables. The Kaplan–Meier log rank method was used for PFS and OS analyses with dichotomized variables (response to NACT and residual tumour in surgery).

## Results

The study sample comprised 23 women, of whom 22 (96%) had been diagnosed with HGSOC and one (4%) with a high-grade endometrioid carcinoma. The patients’ characteristics are presented in Table 2*.* The mean age at the time of diagnosis was 65.6 years (range: 44–78 years). Both the pre- and post-NACT images were available for 17 (74%) of the patients; for six (26%) of the patients, only post-NACT images were available. The median interval between the pre- and post-treatment imaging was 12 weeks (range: 7–22 weeks). Some of the patients had longer imaging intervals due to acute health issues (e.g. pulmonary embolism or acute peritonitis). The median interval between post-treatment-MRI and the IDS attempt was 2 weeks (range: 0–25 weeks). Two (8.7%) of the patients had exceptionally long intervals (20 and 25 weeks) between their post-treatment-MRI scan and the IDS attempt. This was due to acute health issues that had to be treated before cytoreductive surgery and the MDT meetings’ decision to continue chemotherapy for three to four cycles more before the IDS attempt.

**Table 2:** The clinicopathological characteristics of the patients included in the study (*N* = 23)

**Variable**	*n* (%)	***Median (range)***
Age at the time of diagnosis (years)		***68 (44–78) ***
CA-125 (kU/L)		***715 (177–15,495)***
BMI^a^		***25.7 (17.0–38.5)***
FIGO^b^ stage		
IIIC	7 (30)	
IVA	4 (17)	
IVB	12 (52)	
Grade		
Low grade	0 (0)	
High grade	23 (100)	
Histopathology		
Serous carcinoma	22 (96)	
Endometrioid carcinoma	1 (4)	
Debulking operation		
Yes	21 (91)	
No	2 (9)	
Residual tumour at cytoreductive surgery		
0 mm	6 (26)	
≤ 1 cm	6 (26)	
> 1 cm	9 (39)	
Missing (no surgery)^c^	2 (9)	
Neoadjuvant chemotherapy cycles		
3–4	19 (83)	
≥5	4 (17)	
Cancer recurrence within the follow-up period		
No	2 (9)	
Yes	14 (61)	
Progressive disease^d^	7 (30)	
Platinum sensitivity^e^		
Sensitive	***13 (56)***	
Resistant	10 (43)	
Deceased during the follow-up period		
Yes	***20 (87)***	
No	***3 (13)***	

^a^*BMI* Body mass index

^b^Federation of Gynaecology and Obstetrics

^c^Two patients did not undergo surgery because of a lack of a significant treatment response noted in imaging

^d^The patient was defined as having a progressive disease when she received continuous chemotherapy or had an interval of less than 6 months without chemotherapy after the debulking operation

^e^The disease was defined as sensitive to platinum when the patient survived over 6 months without chemotherapy before the possible recurrence, and defined as resistant when the patient received continuous platinum treatment after the intended number of NACT treatments, or the period between NACT and the initiation of chemotherapy was less than 6 months

There were 16 (69.6%) patients with right-sided primary ovarian tumours and seven (30.4%) with left-sided primary ovarian tumours. The ADC measurements were successfully performed by both the observers of pre-NACT/post-NACT scans in 17 out of 18 right ovaries, 14 out of 20 left ovaries, and 14 out of 16 omental metastases. The reasons for the non-successful ADC measurements were poor image quality (right/left ovary *n* = 1/1, omentum *n* = 0); the small size of the tumour, in which the set pixel size of 5×5 pixels for S-ROI would not fit (right/left ovary in pre-NACT images *n* = 0/1 and post-NACT images *n* = 1/3; omentum in pre/post images *n* = 0/5); the absence of the target anatomic structure (absence of the metastatic abdominal mass in the pre-/post-NACT images *n* = 3/5); or non-distinguishable ovaries (right/left ovary in pre-NACT images *n* = 1/3 and post-NACT images *n* = 6/4).

### Intraclass correlations

The interobserver agreement between the two observers was good to excellent for the ovaries, the omentum and the psoas muscle, irrespective of the chosen ROI delineation method. In subcutaneous fat measurements the interobserver agreement was moderate to good, but the fat measurements were not specifically targeted by the two observers. The ICC values for the mean ADC value of the pre-/post-NACT measurements using the L-mean were 0.96/0.96 for the right ovary, 0.95/0.99 for the left ovary, and 0.91/0.98 for the omentum. The corresponding ICC values using the mean ADC value of the S-mean_low_ measurements were 0.96/0.97, 0.96/0.80, and 0.83/0.96, and for S-mean_av._0.96/0.91, 0.83/0.95, and 0.71/0.95, respectively. The ICC values are shown in Table 3*.*

**Table 3:** The interobserver reproducibility of ADC measurements between the two observers using large ROI (L-mean), and the lowest and average mean value obtained using three small ROIs (S-mean_low_ and S-mean_av_)

	**Pre-treatment**			**Post-treatment**		
	***No* of measurements** **Observer 1/2**	**Intraclass correlation coefficient**	**p**	***No* of measurements** **Observer 1/2**	**Intraclass correlation coefficient**	***p***
**Total *N* (patients)**	17			23		
**Right ovary**						
**L-mean**	17 / 17	0.96	< 0.001	18 / 18	0.96	< 0.001
**S-mean**_**low**_	17 / 17	0.96	< 0.001	14 / 16	0.97	< 0.001
***S-mean***_***av***_	*17 / 17*	*0.96*	< *0.001*	*14 / 16*	*0.91*	< *0.001*
**Left ovary**						
**L-mean**	14 / 14	0.95	< 0.001	20 / 20	0.99	< 0.001
**S-mean**_**low**_	13 / 12	0.96	< 0.001	16 / 16	0.80	0.002
***S-mean***_***av***_	*13 / 12*	*0.83*	< *0.001*	*16 / 16*	*0.95*	< *0.001*
**Omentum**						
**L-mean**	14 / 14	0.91	< 0.001	16 / 16	0.98	< 0.001
**S-mean**_**low**_	14 / 14	0.83	0.001	14 / 15	0.96	< 0.001
***S-mean***_***av***_	*14 / 14*	*0.71*	*0.002*	*14 / 15*	*0.95*	< *0.001*
**Psoas muscle**						
**L-mean**	17 / 17	0.67	0.018	23 / 23	0.90	< 0.001
**S-mean**	17 / 17	0.61	0.036	23 / 23	0.83	< 0.001
**Subcutaneous fat**						
**L-mean**	17 / 17	0.75	0.004	23 / 23	0.76	0.001
**S-mean**_**low**_	17 / 17	0.79	0.002	23 / 23	0.68	0.005
*S-mean*_*av*_	*17 / 17*	*0.50*	*0.017*	*23 / 23*	*0.62*	< *0.001*

Only one S-ROI measurement was made in the psoas muscle

## ADC values in the tumoural lesions

**Fig. 2:**
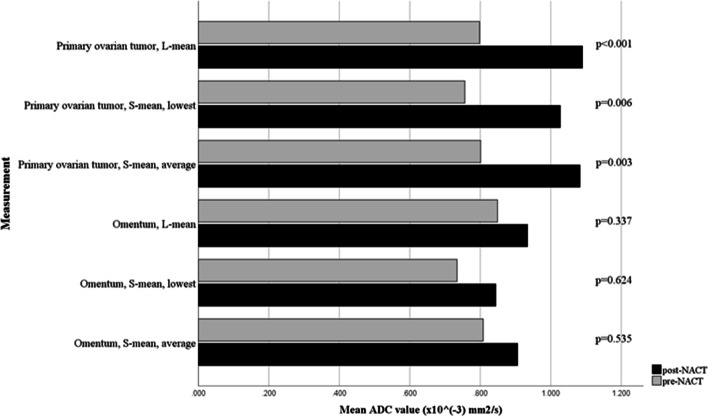
The association between the paired pre- and post-NACT mean ADC values (× 10^–3^ mm^2^/s) in the L-mean, S-mean_low_, and S-mean_av_ measurements

The mean pre- and post-NACT ADC values and percentual ΔADC (ΔADC%) are shown in Table 4, and the changes of mean ADC values are illustrated in Fig. 2. The mean ADC values in the primary ovarian tumour increased significantly between the pre- and post-NACT measurements when using different ROI sampling methods (L-mean ΔADC% = 39.4%, *p* < 0.001; S-mean_low_ ΔADC% = 41.7%, *p* = 0.006; and S-mean_av_ ΔADC% = 40.9%, *p* = 0.003). The minimum ADC values (S-min_low_ and S-min_av_) did not increase significantly between the pre- and post-measurements. The changes in the ADC values in the omental masses were not statistically significant with any of the ROI sampling methods.

**Table 4:** The effect of NACT on pre- and post-treatment mean and minimum ADC values (× 10^–3^ mm^2^/s) in the different locations using large and small ROI

	**Pre-treatment** **ADC**	**Post-treatment** **ADC**	**ΔADC%**	**Pre vs. post NACT change**
	**Mean ± *SD***	**Mean ± *SD***	**%**	***p***
**Primary ovarian tumour**				
**ADC, large ROI, mean**	0.798 ± 0.156	1.090 ± 0.205	39.4	< 0.001
**ADC, small ROI, lowest** ^a^** mean**	0.756 ± 0.195	1.027 ± 0.228	41.7	0.006
**ADC, small ROI, average** ^b^** mean**	0.801 ± 0.176	1.083 ± 0.203	40.9	0.003
**ADC, small ROI, lowest** ^a^** minimum**	0.595 ± 0.195	0.773 ± 0.241	40.5	0.098
**ADC, small ROI, average** ^b^** minimum**	0.634 ± 0.187	0.829 ± 0.238	38.8	0.082
**Omentum**				
**ADC, large ROI, mean**	0.849 ± 0.187	0.934 ± 0.252	10.9	0.337
**ADC, small ROI, lowest** ^a^** mean**	0.734 ± 0.199	0.844 ± 0.286	8.2	0.624
**ADC, small ROI, average** ^b^** mean**	0.808 ± 0.225	0.906 ± 0.270	12.5	0.535
**ADC, small ROI, lowest** ^a^** minimum**	0.523 ± 0.204	0.654 ± 0.274	8.2	0.509
**ADC, small ROI, average** ^b^** minimum**	0.614 ± 0.209	0.735 ± 0.254	12.5	0.451
**Psoas muscle**				
**ADC, large ROI**	1.171 ± 0.129	1.238 ± 0.157	5.7	0.237
**ADC, small ROI, lowest**^a^	1.120 ± 0.148	1.216 ± 0.229	10.1	0.156
**Subcutaneous fat**				
**ADC, large ROI**	0.854 ± 0.359	0.734 ± 0.343	-3.5	0.422
**ADC, small ROI, lowest**^a^	0.756 ± 0.360	0.625 ± 0.341	-8.5	0.328

Number of measurements is shown in Table 3 (Observer 1)

^a^Small ROI, lowest = the lowest mean or minimum ADC value obtained using three small ROIs

^b^Small ROI, average = the average mean or minimum ADC value obtained using three measured small ROIs

When the ADC values of the primary ovarian tumours were compared with those of the omentum, no significant differences were found in the pre-NACT L-ROI or any of the S-ROI measurements. The post-NACT ADC values of the primary ovarian tumour were higher than those of the omentum when using the mean ADC values; however, the result was statistically significant only when the S-mean_low_ was used (primary tumour vs. omentum: S-mean_low_ *p* = 0.017, L-mean *p* = 0.075, and S-mean_av_ *p* = 0.059). Again, the minimum ADC values (S-min_low_ and S-min_av_) did not increase significantly between the pre- and post-measurements.

### Association between the ADC values and the prognostic factors of OC

**Fig. 3:**
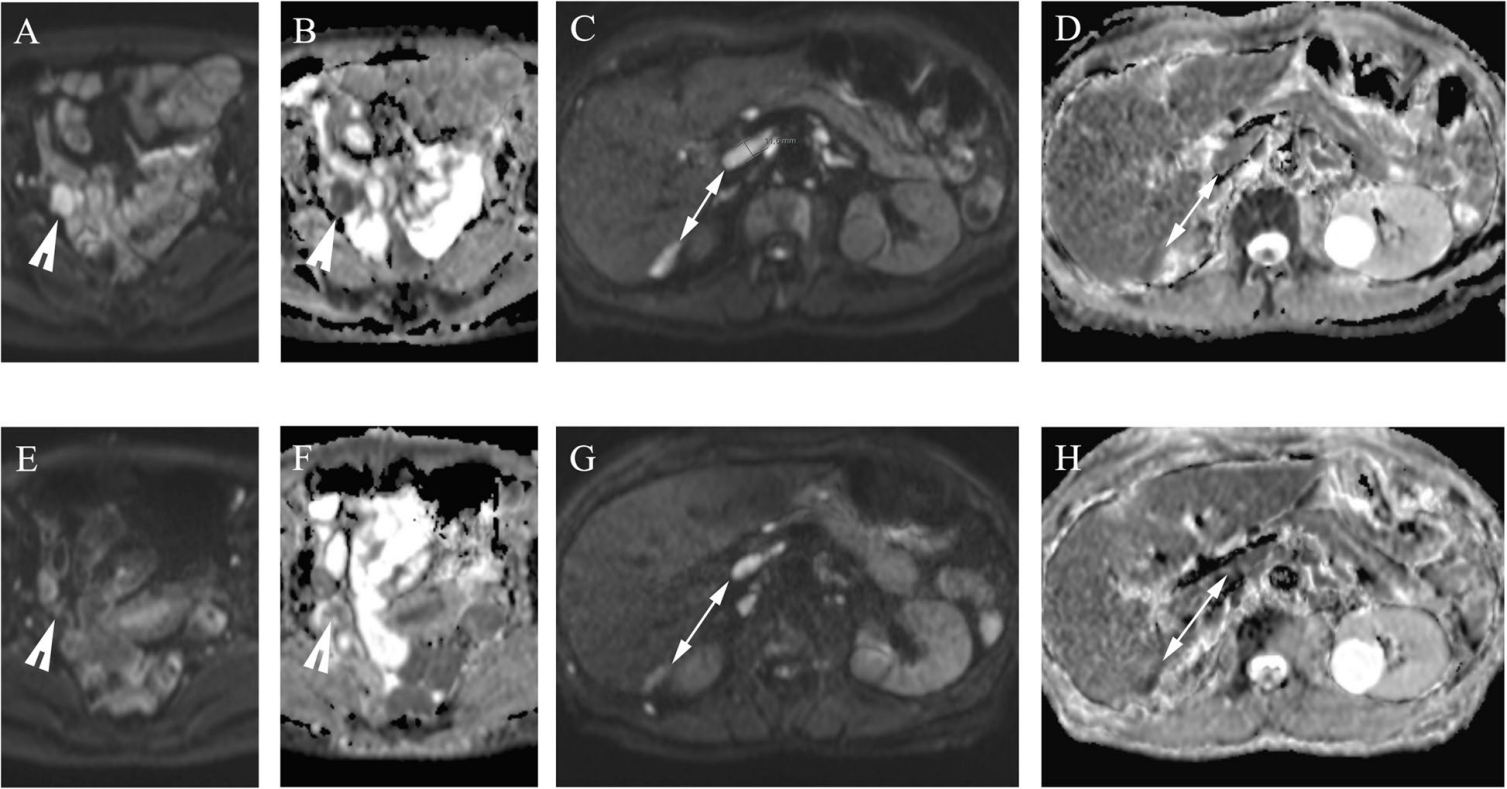
The ovarian and omental pre- and post-treatment ADC maps used in the evaluation of the response to NACT in epithelial OC. A woman in her late 60 s was diagnosed with metastatic high-grade serous OC and initially treated with three cycles of NACT. At the time of diagnosis, the right ovary (arrowhead) was hyperintense in the DW image with a high *b*-value of 800 s/mm^2^ (**A**) and displayed a diffusion restriction in the ADC map with a mean ADC value of 0.744 × 10^–3^ mm^2^/s measured with a large ROI (**B**). Correspondingly, the metastatic sites (two-headed arrow) located in the upper abdomen in a high *b*-value DW image were hyperintense (**C**) and showed diffusion restriction on the ADC maps (**D**), with a mean ADC value of 0.744 × 10^–3^ mm^2^/s. After three cycles of NACT, the right ovary had shrunk, as reflected in the high *b*-value image (**E**) and the mean ADC value was elevated to 1.374 × 10^–3^ mm^2^/s (**F**). A similar response was noted in the left ovary (not shown). The abdominal metastatic sites did not respond markedly in volume (**G**). However, in the ADC map (**H**), the mean ADC value had increased to 1.042 × 10^–3^ mm^2^/s. In the response evaluation, the advanced OC did not respond sufficiently to NACT and, thus, there was no attempt to perform interval debulking surgery for this patient. The chemotherapy was continued instead

**Fig. 4:**
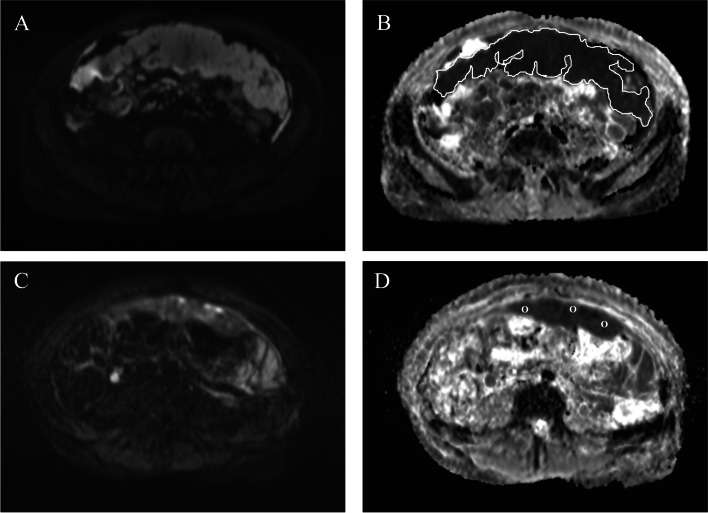
Pre- and post-treatment images of a patient in her late 60 s with metastatic ovarian cancer.The metastatic omentum appears bright in the axial DW image (*b*-value of 800 s/mm^2^), taken at the time of the diagnosis (**A**). An L-ROI was drawn around the omentum on the ADC map generated with *b*-values of 50 s/mm^2^, 400 s/mm^2^, and 800 s/mm^2^ (**B**). The omentum showed restricted diffusion with a mean ADC value of 0.999 × 10^–3^ mm^2^/s because of increased cellularity of the tumour. After four NACT cycles, the high-signal omentum had shrunk in volume (**C**). Three small round ROIs are shown inside the diminished omental mass in the ADC map, with the minimum ADC values in the range between 0.693–0.799 × 10^–3^ mm^2^/s (**D**)

**Fig. 5:**
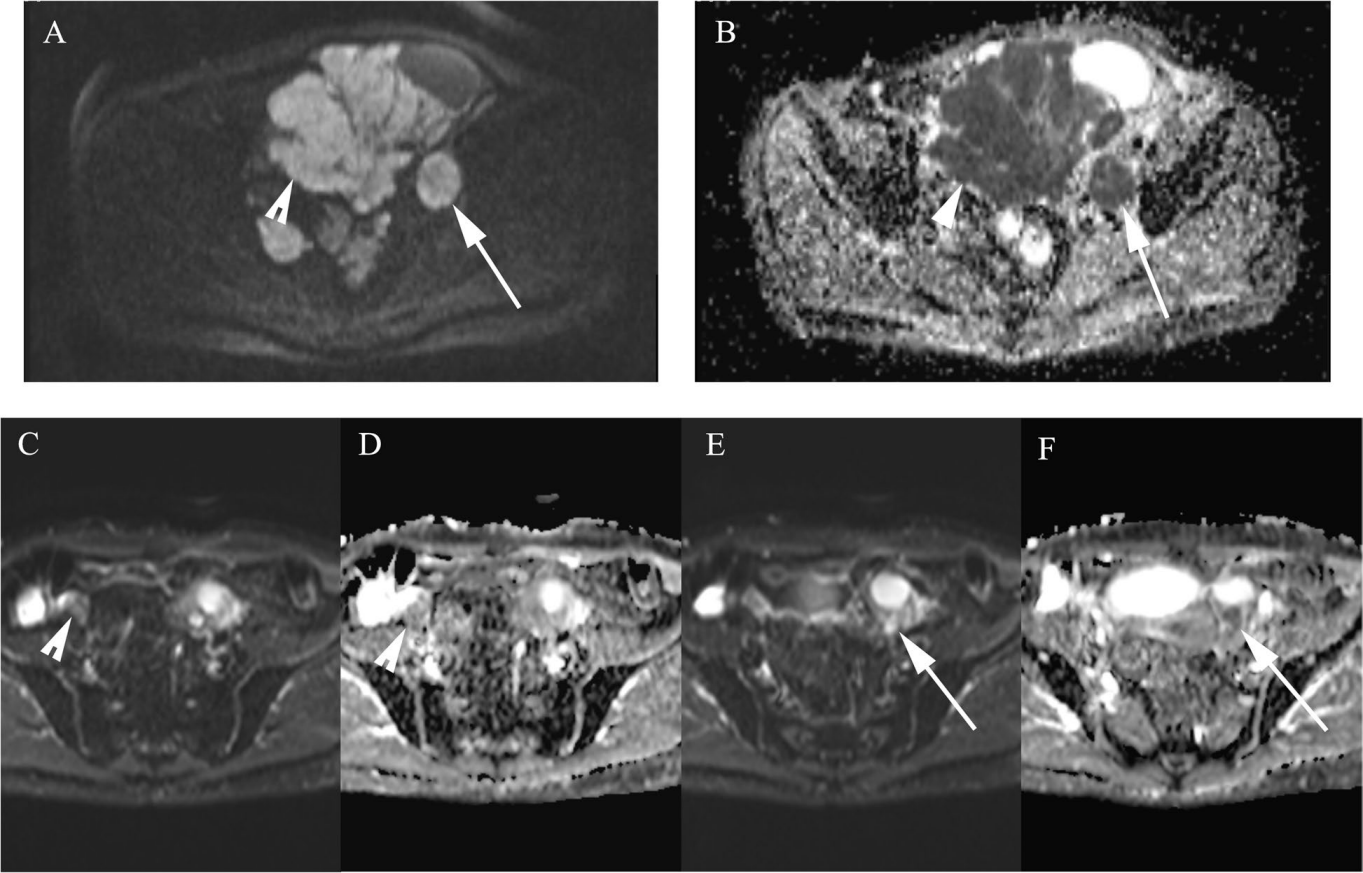
Illustrative pre- and post-treatment images of a woman in her mid-50 s with high-grade serous epithelial OC*.* At the time of diagnosis, the primary tumour in the right ovary (arrowhead) and the left ovary (arrow) displayed a high signal in the DWI-sequence (*b*-value = 800 s/mm^2^) (**A**) and restricted diffusion with mean ADC values of 0.625 × 10^–3^ mm^2^/s and 0.694 × 10^–3^ mm^2^/s, respectively (**B**). After four cycles of NACT, the right ovary (arrowhead) was smaller in the high *b*-value images (**C**) and the diffusion restriction was less intense in the ADC map, with the mean ADC value increasing to 1.532 × 10^–3^ mm^2^/s (**D**). A similar positive response was seen in the left ovary (arrow) in the DWIBS-image (DW imaging with background body signal suppression) (**E**) and ADC map, with a mean ADC value of 1.134 × 10^–3^ mm.^2^/s (**F**)

Two (8.7%) patients did not continue to IDS based on the MDT evaluation and continued with chemotherapy (Fig. 3). The other 21 (91.3%) patients responded radiologically to NACT and underwent an IDS attempt after NACT (Figs. 4 and 5). Based on the MDT evaluation and the report from surgery, 13 (56.5%) patients were defined as responders to NACT and 10 (43.5%) (including the two patients who did not proceed to IDS) as non-responders.

**Fig. 6:**
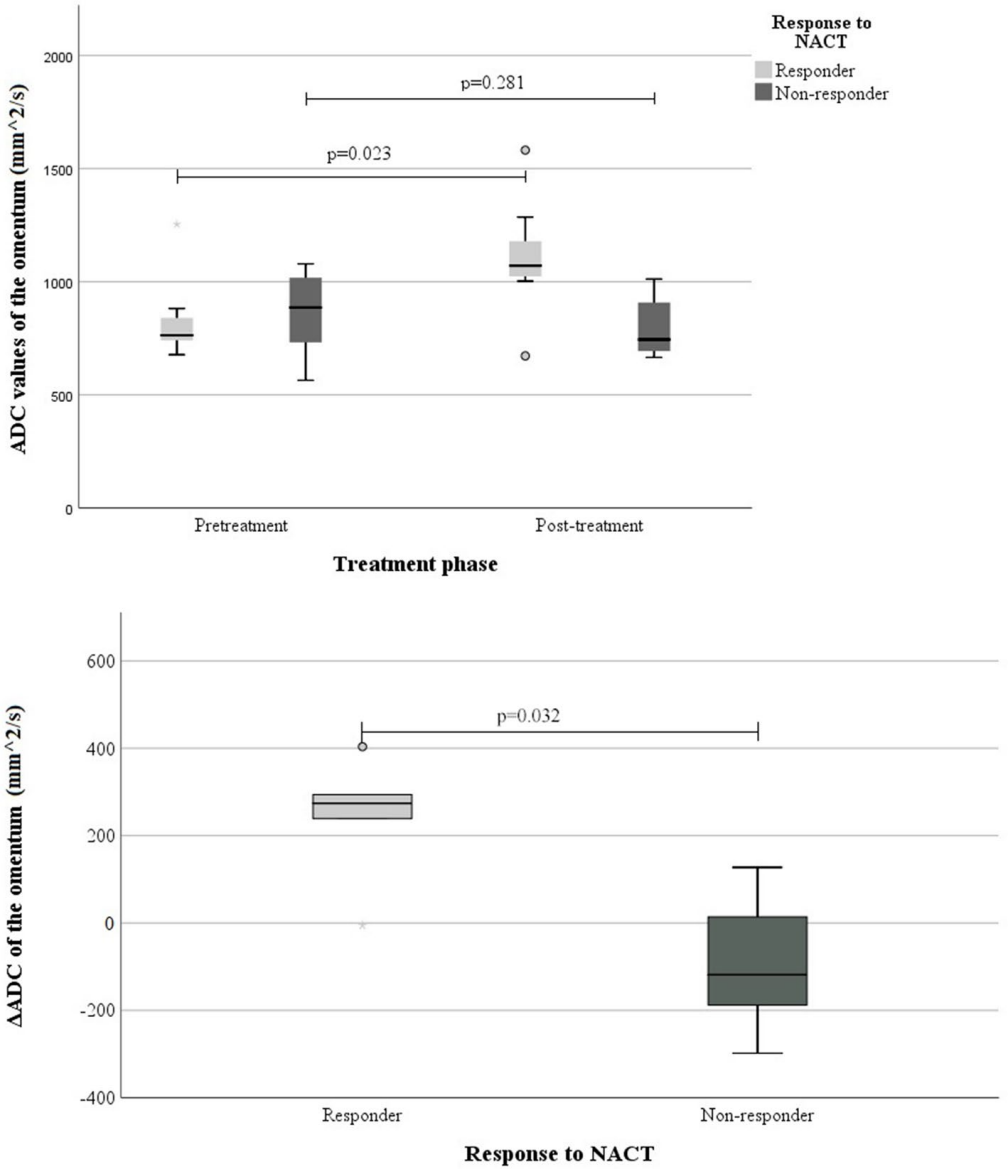
The association between the response to neoadjuvant chemotherapy and the mean ADC values in the omental metastasis (L-mean) The upper figure indicates the pre- and post-treatment ADC values separately and the difference between the two groups, responders and non-responders. The lower figure indicates the change between pre- and post-treatment ADC values and the difference between responders and non-responders. The boxes indicate the median and the quartiles 2- and 3 of the ADC values, the whiskers indicate the lowest and the highest data points, and the dots above or below the whiskers indicate the outliers

The response to chemotherapy was significantly associated with the L-mean ΔADC and ΔADC% in the omental metastasis (ΔADC/ΔADC% *p* = 0.032/0.032, *n* = 10). Small ROI measurements or the measurements of the primary ovarian tumour did not have a significant association with the response to NACT. The change between tumoural pre- and post-treatment ADC values was greater in responders than in non-responders in the omental metastasis. The mean pre- and post-NACT ADC values and the percentual ΔADC (ΔADC%) are shown in Table 5, and the changes in the mean ADC values are illustrated in Fig. 6.

**Table 5:** The association between the response to NACT and the difference between pre- and post-treatment mean ADC values (× 10^–3^ mm^2^/s) in omental metastasis

**Response to NACT**^a^	**Pre-treatment ADC in omental metastasis**	**Post-treatment ADC in omental metastasis**	**ΔADC**	**ΔADC%**	*p* **
(*n*)	Mean ± *SD*	Mean ± *SD*	Mean ± *SD*	%	ΔADC / ΔADC% (n)
**Responder**					L-mean *p* = 0.032 / 0.032 (10) S-mean_low_* p* = 0.143 / 0.250 (8) S-mean_av_ *p*= 0.143 / 0.143 (8)
ADC, L-mean (5)	0.836 ± 0.195	1.103 ± 0.278	0.241 ± 0.184	30.0	
ADC, S-mean_low_ (3)	0.721 ± 0.235	1.031 ± 0.378	0.214 ± 0.183	29.6	
ADC, S-mean_av_ (3)	0.801 ± 0.274	1.106 ± 0.346	0.278 ± 0.187	38.5	
**Non-responder**					
ADC, L-mean (5)	0.861 ± 0.194	0.802 ± 0.130	-0.093 ± 0.167	-8.1	
ADC, S-mean_low_ (5)	0.746 ± 0.171	0.740 ± 0.165	-0.064 ± 0.182	-4.6	
ADC, S-mean_av_ (5)	0.815 ± 0.188	0.795 ± 0.141	-0.070 ± 0.214	-3.0	

^a^Response to platinum-based NACT was defined by combining the results of radiological response evaluation and the approximate amount of visible tumour or carcinosis left after the interval debulking surgery

** P-value indicates the association between the dichotomous response to NACT (responder vs. non-responder) and the ΔADC and ΔADC% values in the omental metastasis

**Fig. 7:**
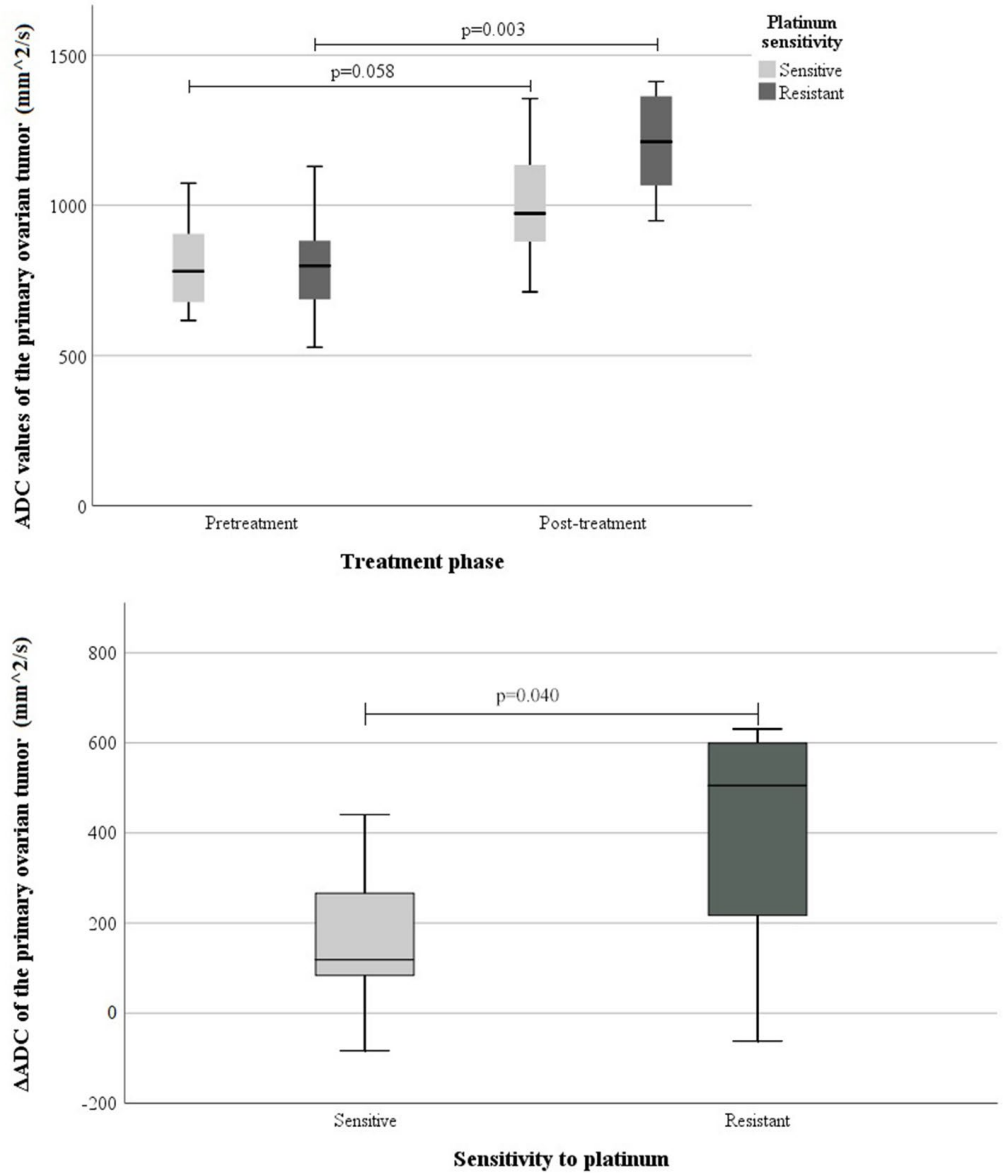
The association between sensitivity to platinum-based chemotherapy and the mean ADC value in the primary ovarian tumour (L-mean) The upper figure indicates the pre- and post-treatment ADC values separately and the difference between the two groups (platinum sensitive and platinum resistant). The lower figure indicates the change between pre- and post-treatment ADC values and the difference between the two groups (platinum sensitive and platinum resistant). The boxes indicate the median and quartiles 2- and 3 of the ADC values, and the whiskers indicate the lowest and the highest data points

The dichotomized platinum sensitivity (sensitive or resistant) was significantly associated with the ΔADC and ΔADC% in the primary ovarian tumour (ΔADC/ΔADC% L-mean *p* = 0.040/0.072, S-mean_low_ *p* = 0.018/0.030, S-mean_av_ *p* = 0.048/0.048). Patients who had a cancer recurrence in less than 6 months, namely those with platinum-resistant tumours, had greater (percentual) difference between pre- and post-NACT ADC values than those with tumours sensitive to platinum. The mean pre- and post-NACT ADC values and percentual ΔADC (ΔADC%) are shown in Table 6, and the changes of mean ADC values are illustrated in Fig. 7. Neither the pre- nor the post-NACT ADC values or ΔADC were significantly associated with the categorized clinical variables (i.e., cancer recurrence or the presence of macroscopic residual tumour in the operation).

**Table 6:** The association between the sensitivity to platinum-based chemotherapy and the difference between pre- and post-treatment mean ADC values (× 10^–3^ mm^2^/s) in the primary ovarian tumour

Sensitivity to platinum	Pre-treatment ADC in primary tumour	Post-treatment ADC in primary tumour	ΔADC	ΔADC%	p***
(*n*)	Mean ± *SD*	Mean ± *SD*	Mean ± *SD*	%	ΔADC / ΔADC% (*n*)
**Sensitive or semi-sensitive**^a^					L-mean *p* = 0.040 / 0.072 (15) S-mean_low_ *p* = 0.018 / 0.030 (12) S-mean_av_ *p* = 0.048 / 0.048 (12)
ADC, L-mean (7)	0.802 ± 0.156	0.991 ± 0.189	0.168 ± 0.190	23.9	
ADC, S-mean_low_ (5)	0.771 ± 0.214	0.929 ± 0.232	0.052 ± 0.221	10.9	
ADC, S-mean_av_(5)	0.815 ± 0.185	0.978 ± 0.0.185	0.080 ± 0.206	13.4	
**Resistant**^b^					
ADC, L-mean (8)	0.795 ± 0.168	1.199 ± 0.170	0.401 ± 0.251	53.0	
ADC, S-mean_low_ (7)	0.743 ± 0.188	1.112 ± 0.199	0.431 ± 0.194	63.6	
ADC, S-mean_av_ (7)	0.790 ± 0.178	1.175 ± 0.180	0.442 ± 0.209	60.5	

^a^Over 6 months without platinum-based chemotherapy before cancer recurrence

^b^Less than 6 months without platinum-based chemotherapy before cancer recurrence

*** P-value indicates the association between the dichotomous sensitivity to platinum chemotherapy (sensitive/semi-sensitive vs. resistant) and the ΔADC and ΔADC% values in the primary ovarian tumour

The CA-125 values were inversely correlated with the pre-NACT values of the omentum (*r*_*s*_ = -0.644/-0.578, *p* = 0.013/0.030 for S-mean_av_/S-mean_low_) and with the post-NACT values of the primary ovarian tumour (S-min_low_ *r*_*s*_ = -0.529, *p* = 0.043). The continuous clinical markers age and BMI did not significantly correlate with the ADC measurements of the tumoural lesions.

### Patient survival

The median follow-up time was 36 months (range: 7–114 months). Two patients out of 23 (9%) with a follow-up of 9.5 and 7.5 years had no cancer recurrence by the end of the follow-up period (from the time of diagnosis until January 20^th^ 2023). Fourteen patients (61%) had a cancer recurrence, and seven patients (30%) were diagnosed with having a progressive disease by means of imaging. At the end of the follow-up, three patients (13%) were alive and 20 (87%) were deceased. Two (8.7%) patients died during the first year of follow-up (OS in 1 year) and the OS rate was 11 (47.8%) and 14 (60.9%) at the 2- and 3-year timepoints, respectively. At the 1-year timepoint, 17 (73.9%) patients had had cancer recurrence or progression (PFS in 1 year). The PFS for the 2- and 3-year timepoints was 21 (91.3%) and 21 (91.3%), respectively. The median PFS was 9 months (range: 0–111 months) and OS 31 months (range: 2–111 months).

The response to NACT correlated with the 2- and 3-year OS (*p* = 0.038 and *p* = 0.009, respectively) and the 1-, 2- and 3- year PFS (*p* = 0.006, *p* = 0.006 and *p* = 0.006, respectively. The amount of residual tumour in surgery (ie. presence of residual tumour in surgery or no visible residual disease) correlated with the 2- and 3-year OS (*p* = 0.007 and *p* = 0.011, respectively) and with the 1-, 2- and 3-year PFS (*p* = 0.002, *p* = 0.001 and *p* = 0.001, respectively).

Neither pre- or post-NACT ADC values nor the higher ΔADC predicted patients’ overall survival or recurrence-free survival.

## Discussion

Quantitative analysis of ADC maps in OC has previously shown promise in diagnostic and prognostic assessments, as well as in predicting NACT treatment response [17, 23, 26–29]. However, the integration of ADC measurements into clinical practice requires further research to define the most robust method for performing quantitative analyses in terms of performance and reproducibility, and to determine the optimal cut-off values for ADC change [18, 25, 30]. We retrospectively enrolled 23 patients with OC to examine whether the possible increase of ADC values could predict the response to NACT treatment. As there is still no clear consensus on the optimal way to perform ADC measurements in OC, we also compared several ROI sampling methods. Our results showed a significant increase of the mean ADC values in the primary tumour mass in the ovary between the pre- and post-treatment measurements when using both large and small ROIs. Interestingly, the platinum-resistant tumours showed greater change and higher post-treatment ADC values than the platinum-sensitive ones. In the omental metastasis, in contrast, the change between the pre- and post-treatment large ROI measurements was significantly greater in patients who responded well to NACT than in non-responders. In our cohort, with its limited number of patients, ADC measurements did not predict recurrence-free or overall survival.

After transvaginal ultrasound, CT is the standard imaging modality to investigate the possibility of OC. Its key role is the identification of disease bulk beyond the lesion and metastasis to other organ systems, both of which could lead to more radical interventions [31, 32]. Multiparametric MRI is increasingly being exploited in routine clinical practice. Low pre-treatment ADC values in primary OC might be predictive of OC histopathology, such as poorly differentiated tumours. They could therefore indicate a lower chance of survival [19]. Pre-treatment ADC histogram analysis has also been found to be a promising approach when planning chemotherapy by predicting advanced OC patients’ responses to platinum-based chemotherapy [27]. Novel tumour characterization models are also being investigated and integrative MRI- and CT-based machine learning models have been proposed [33–36].

The optimal parameters and ROI selection method warrant further research. As far as we are aware, there is no discussion in the literature about which ROI method should be selected for the evaluation of the NACT response in OC or whether the ROI sampling method has clinical significance. Indeed, unlike for breast [37, 38] and rectal [39] cancers, no standardized method for performing the ADC measurements exists for ovarian tumours. Mukuda et al. [24] demonstrated that the freehand and round-shaped ROIs have greater interobserver reliability than square ROIs in terms of differential diagnostics of ovarian tumours by mean ADC values. Similar to this finding, we demonstrated that both a protocol with a large L-ROI drawn free-hand on a single slice along the tumour border, avoiding cystic areas, and a fixed-sized S-ROI (5× 5 pixels) also exhibited good to excellent interobserver agreement in patients treated with NACT. Furthermore, our results indicate that the use of mean ADC values, rather than minimum ADC values, gives more reproducible results when imaging heterogeneous ovarian tumours. However, in agreement with Mukuda et al., we noted that minimum and mean ADC values might vary significantly, which could lead to a misinterpretation of ADC values as a biomarker.

In the current study, the ADC values increased more after NACT for the platinum-resistant tumours than for the platinum-sensitive ones. In contrast, the ADC values of the omental metastasis increased more for the patients who responded well to NACT than for non-responders. We hypothesize that the difference between the NACT response in the primary ovarian tumour and the omental metastasis might well arise from the histopathological heterogeneity of OC. Differences in the ADC values between the primary ovarian tumour and metastatic deposits have previously been demonstrated [40] and our results are consistent with this finding. In our small sample, the omental ADC values increased significantly in responders, and in non-responders the values diminished or remained the same, as expected. In the primary ovarian tumours, the changes were not significant in responders or in non-responders. One can speculate that the primary tumour in the ovary might react to chemotherapy in a different manner than the metastatic lesion. In our study, the tumoural lesions’ varied responses to NACT might also be explained by the small population size. Larger cohorts are needed to confirm this finding.

Our finding of a significant increase in the mean ADC values after platinum-based NACT is consistent with previous studies [23, 26]. Winfield et al. [23] reported, on the basis of a sample of 125 patients who had advanced OC (47 primary and 78 relapsed), that three cycles of NACT induced a significant change in ovarian ADC values. The change was greater in responders than in non-responders (biochemical response definition; thus, reduction of 50% in CA-125 *p* = 0.02; radiologic response definition using Response Evaluation Criteria in Solid Tumours [RECIST] *p* = 0.04). The omental metastases were not evaluated and the median ADC value was measured by a ROI drawn on every slice in which a lesion appeared. Kyriazi et al. [26] detected a significant increase in ADC values and a decrease in the size of the primary tumours and omental metastases after patients with advanced ovarian or primary peritoneal cancers (*n* = 8) had undergone one and three cycles of NACT (*p* < 0.001). The finding was associated with a partial response to NACT when evaluated by the tumour shrinkage in cross-sectional imaging. The ROI was delineated with interactive computer-assisted segmentation, where the operator pointed the target lesion, and the mean ADC value of the whole tumour volume was used. The baseline ADC values did not differ between the ovarian tumour and omental lesion, which is consistent with our results. In contrast to the work of Kyriazi et al., only the ovarian ADC values increased after NACT in the current study; there was no statistical difference in the omental ADC values between the pre- and post-NACT scans. Furthermore, similarly to our findings, tumoural ADC values have been reported to be elevated in response to NACT in other cancers such as breast, cervical, and rectal cancers [41–45].

The association between mean ADC values and the response to platinum-based chemotherapy in advanced OC was shown  in a previous study by Lu et al. [27], indicating that lower mean ADC values of the primary ovarian tumour in pre-treatment imaging are more likely to be platinum-resistant. In this study, the difference between the pre- and post-NACT ADC values (ΔADC) in the primary ovarian tumour was associated with sensitivity to platinum-based chemotherapy. The ΔADC was greater and the post-NACT ADC values were slightly higher for tumours that were platinum-resistant. The pre-treatment values did not differ significantly between the two groups. Our finding indicates that the change between pre- and post-treatment ADC values, not the values themselves, is significant in evaluating the platinum sensitivity of heterogeneous ovarian tumours.

The response to NACT and the presence of residual tumour in the interval debulking surgery was associated with the OS in 2- and 3-year timepoints and the PFS with 1-, 2- and 3-year timepoints. This is consistent with previous studies showing that the  amount of residual tumour in cytoreductive surgery (both PDS and IDS) is one of the most significant factors associated with the survival rates in OC patients [9, 11] and that the response to NACT has an impact on OS and PFS [46]. There were no other statistically significant relationships between pre- and post-NACT ADC-values and patient survival or clinical cancer markers. This is probably partly attributable to the small size of our cohort and the relatively brief follow-up period of some of the enrolled patients.

Our study has several limitations. There were only 23 patients, and both pre- and post-NACT MR images were available for only 17 of them. The cohort comprised patients with the most common type of OC, the epithelial type, which is treated similarly regardless the histopathological subtype [47]. Our small sample illustrates well the rarity of advanced OCs treated with NACT. Larger cohorts are needed to evaluate the clinical significance of the NACT-induced ADC increase in OC with respect to radiological response to NACT and survival benefit. Moreover, as the study was planned retrospectively, the imaging protocols were not standardized. However, all the patients were scanned with DWI protocols that included low and high *b*-value images (Table 1) and it was therefore possible to demonstrate the diffusion restriction clearly in every case, provided that there was a residual tumour that allowed for measurement. The non-standardized imaging protocols may also lead to variation in small ROIs area sizes when using pixel defined size. However, we wanted to use a tool which is both practical and easily reproducible in clinical practice. Furthermore, the patients were imaged using different scanners (1.5 T and 3.0 T scanners by two manufacturers). This is a challenge in retrospective studies with no harmonization of MRI data, because even switching between MRI scanners from the same manufacturer may lead to non-biological variance in ADC values [48, 49]***.*** However, there was no cross-over between field strengths. In addition, the number of NACT cycles or the post-treatment imaging timing was not standardized. Although these factors might have affected the ADC values, this is unlikely to affect our conclusion regarding interobserver reproducibility.

## Conclusion

DW imaging and a quantitative determination of the ADC value possess diagnostic potential in the characterization and evaluation of NACT-treatment response in patients with advanced OC. When measured with either large freehand or small, round ROI on a single slice, the tumoural mean ADC values were significantly higher in the primary ovarian tumour after NACT than before NACT. The minimum ADC values did not change significantly. The large freehand measurements were also associated with the response to NACT and the sensitivity to platinum-based chemotherapy. Our study results support the use of single-plane L-ROIs when determining any changes in the mean ADC values in response to NACT, which is a simple method that is suitable for clinical use. Larger cohorts are needed to evaluate the role of ADC measurements in the treatment decisions of individual OC patients and to standardize a ROI delineation method for ADC measurements.

## Data Availability

The datasets used and analysed during the study are available from the corresponding author on reasonable request.

## Abbreviations

ADC: Apparent diffusion coefficient
ΔADC: The change of ADC values during the NACT treatment
ΔADC%: The percentual ΔADC
L-mean: The mean ADC value of large ROI measurements
S-mean_low_: The lowest mean ADC value of one to three measured small ROI measurements
S-mean_av_: The average mean ADC value of one to three measured small ROI measurements
S-min_low_: The lowest minimum ADC value of one to three measured small ROI measurements
S-min_av_: The average mean ADC value of one to three measured small ROI measurements
ACR: American College of Radiology
ASCO: American Society of Clinical Oncology
BMI: Body mass index
CA-125: Cancer antigen 12–5
CT: Computed tomography
DCE: Dynamic contrast-enhanced sequences
DWI: Diffusion-weighted imaging
ESGO: European Society of Gynaecological Oncology
ESMO: European Society of Medical Oncology
ESUR: European Society of Urogenital Radiology
FIGO: Federation of Gynaecology and Obstetrics
HGSOC: High-grade serous ovarian cancer
ICC: Interclass correlation coefficient
IDS: Interval debulking surgery
MDT: Multi-disciplinary team
MRI: Magnetic resonance imaging
mMRI: Multiparametric MRI
NACT: Neoadjuvant chemotherapy
NCCN: National Comprehensive Cancer Network
OC: Ovarian cancer
OS: Overall survival
PDS: Primary debulking surgery
PFS: Progression-free survival
ROI: Region of interest
L-ROI: Large freehand ROI
S-ROI: Small round ROI
SGO: Society of Gynecologic Oncology
TE: Echo time
TR: Repetition time
T1W: T1-weighted imaging
T2W: T2-weighted imaging

## References

[1] Sung H, Ferlay J, Siegel RL, Laversanne M, Soerjomataram I, Jemal A (2021). Global Cancer Statistics 2020: GLOBOCAN Estimates of Incidence and Mortality Worldwide for 36 Cancers in 185 Countries. CA Cancer J Clin.

[2] Lheureux S, Gourley C, Vergote I, Oza AM (2019). Epithelial ovarian cancer. Lancet.

[3] Forstner R (2020). Early detection of ovarian cancer. Eur Radiol.

[4] Berek JS, Renz M, Kehoe S, Kumar L, Friedlander M (2021). Cancer of the ovary, fallopian tube, and peritoneum: 2021 update. Int J Gynecol Obstet.

[5] Zhou J, Wu SG, Wang J, Sun JY, He ZY, Jin X (2018). The effect of histological subtypes on outcomes of stage IV epithelial ovarian cancer. Front Oncol.

[6] Yang SP, Su HL, Chen XB, Hua L, Chen JX, Hu M (2021). Long-term survival among histological subtypes in advanced epithelial ovarian cancer: Population-based study using the surveillance, epidemiology, and end results database. JMIR Public Heal Surveill.

[7] Timmerman D, Ameye L, Fischerova D, Epstein E, Melis GB, Guerriero S (2010). Simple ultrasound rules to distinguish between benign and malignant adnexal masses before surgery: Prospective validation by IOTA group. BMJ.

[8] Nishio S, Ushijima K (2020). Clinical significance of primary debulking surgery and neoadjuvant chemotherapy-interval debulking surgery in advanced ovarian cancer. Jpn J Clin Oncol.

[9] Du Bois A, Reuss A, Pujade-Lauraine E, Harter P, Ray-Coquard I, Pfisterer J (2009). Role of surgical outcome as prognostic factor in advanced epithelial ovarian cancer: A combined exploratory analysis of 3 prospectively randomized phase 3 multicenter trials: by the arbeitsgemeinschaft gynaekologische onkologie studiengruppe ovarialkarzin. Cancer.

[10] Armstrong DK, Alvarez RD, Bakkum-Gamez JN, Barroilhet L, Behbakht K, Berchuck A (2019). Ovarian cancer, version 1.2019 Featured Updates to the NCCN Guidelines. JNCCN J Natl Compr Cancer Netw.

[11] Vergote I, Tropé CG, Amant F, Kristensen GB, Ehlen T, Johnson N (2010). Neoadjuvant Chemotherapy or Primary Surgery in Stage IIIC or IV Ovarian Cancer. N Engl J Med.

[12] Kehoe S, Hook J, Nankivell M, Jayson GC, Kitchener H, Lopes T (2015). Primary chemotherapy versus primary surgery for newly diagnosed advanced ovarian cancer (CHORUS): An open-label, randomised, controlled, non-inferiority trial. Lancet [Internet].

[13] Onda T, Satoh T, Saito T, Kasamatsu T, Nakanishi T, Nakamura K (2016). Comparison of treatment invasiveness between upfront debulking surgery versus interval debulking surgery following neoadjuvant chemotherapy for stage III/IV ovarian, tubal, and peritoneal cancers in a phase III randomised trial: Japan Clinical Oncology Group Study JCOG0602. Eur J Cancer [Internet].

[14] Colombo N, Sessa C, Du Bois A, Ledermann J, McCluggage WG, McNeish I (2019). ESMO-ESGO consensus conference recommendations on ovarian cancer: Pathology and molecular biology, early and advanced stages, borderline tumours and recurrent disease. Ann Oncol.

[15] Querleu D, Planchamp F, Chiva L, Fotopoulou C, Barton D, Cibula D (2017). European society of Gynaecological Oncology (ESGO) guidelines for ovarian cancer surgery. Int J Gynecol Cancer.

[16] Forstner R, Thomassin-Naggara I, Cunha TM, Kinkel K, Masselli G, Kubik-Huch R (2017). ESUR recommendations for MR imaging of the sonographically indeterminate adnexal mass: an update. Eur Radiol.

[17] Kyriazi S, Collins DJ, Morgan VA, Giles SL, deSouza NM (2010). Diffusion-weighted imaging of peritoneal disease for noninvasive staging of advanced ovarian cancer. Radiographics.

[18] Rizzo S, Del Grande M, Manganaro L, Papadia A, Del Grande F (2020). Imaging before cytoreductive surgery in advanced ovarian cancer patients. Int J Gynecol Cancer.

[19] Lindgren A, Anttila M, Rautiainen S, Arponen O, Kivelä A, Mäkinen P (2017). Primary and metastatic ovarian cancer: Characterization by 3.0T diffusion-weighted MRI. Eur Radiol.

[20] Lindgren A, Anttila M, Arponen O, Rautiainen S, Könönen M, Vanninen R (2019). Prognostic Value of Preoperative Dynamic Contrast-Enhanced Magnetic Resonance Imaging in Epithelial Ovarian Cancer. Eur J Radiol.

[21] Addley H, Moyle P, Freeman S (2017). Diffusion-weighted imaging in gynaecological malignancy. Clin Radiol.

[22] Padhani AR, Miles KA (2010). Multiparametric Imaging of Tumor Response to Therapy. Radiology.

[23] Winfield JM, Wakefield JC, Dolling D, Hall M, Freeman S, Brenton JD (2019). Diffusion-weighted MRI in Advanced Epithelial Ovarian Cancer: Apparent Diffusion Coefficient as a Response Marker. Radiology.

[24] Mukuda N, Fujii S, Inoue C, Fukunaga T, Tanabe Y, Itamochi H (2016). Apparent Diffusion Coefficient (ADC) Measurement in Ovarian Tumor: Effect of Region-of-Interest Methods on ADC values and Diagnostic Ability. J Magn Reson Imaging.

[25] Lu JJ, Pi S, Ma FH, Zhang GF, Wei QJ (2019). Apparent diffusion coefficients measured using different regions of interest in differentiating borderline from malignant ovarian tumors. Acta radiol.

[26] Kyriazi S, Nye E, Stamp G, Collins DJ, Kaye SB, Desouza NM (2010). Value of diffusion-weighted imaging for assessing site-specific response of advanced ovarian cancer to neoadjuvant chemotherapy: Correlation of apparent diffusion coefficients with epithelial and stromal densities on histology. Cancer Biomark.

[27] Lu J, Li HM, Cai SQ, Zhao SH, Ma FH, Li YA (2021). Prediction of Platinum-based Chemotherapy Response in Advanced High-grade Serous Ovarian Cancer: ADC Histogram Analysis of Primary Tumors. Acad Radiol.

[28] Vargas HA, Barrett T, Sala E (2013). MRI of ovarian masses. J Magn Reson Imaging.

[29] Singla V, Dawadi K, Singh T, Prabhakar N, Srinivasan R, Suri V (2021). Multiparametric MRI Evaluation of Complex Ovarian Masses. Curr Probl Diagn Radiol.

[30] Pi S, Cao R, Qiang JW, Guo YH (2018). Utility of DWI with quantitative ADC values in ovarian tumors : a meta-analysis of diagnostic test performance. Acta radiol.

[31] Rockall AG, Qureshi MPI (2016). Role of Imaging in Fertility-sparing Treatment of Gynecologic Malignacies. Radiographics.

[32] Kyriazi S, Kaye SB, Desouza NM (2010). Imaging ovarian cancer and peritoneal metastases - current and emerging techniques. Nat Rev Clin Oncol.

[33] Lei R, Yu Y, Li Q, Yao Q, Wang J, Gao M (2022). Deep learning magnetic resonance imaging predicts platinum sensitivity in patients with epithelial ovarian cancer. Front Oncol.

[34] Boehm KM, Aherne EA, Ellenson L, Nikolovski I, Alghamdi M, Vázquez-García I (2022). Multimodal data integration using machine learning improves risk stratification of high-grade serous ovarian cancer. Nat Cancer.

[35] Avesani G, Tran HE, Cammarata G, Botta F, Raimondi S, Russo L (2022). CT-Based Radiomics and Deep Learning for BRCA Mutation and Progression-Free Survival Prediction in Ovarian Cancer Using a Multicentric Dataset. Cancers (Basel).

[36] Wang X, Li H, Zheng P (2022). Automatic Detection and Segmentation of Ovarian Cancer Using a Multitask Model in Pelvic CT Images. Oxid Med Cell Longev.

[37] Arponen O, Sudah M, Masarwah A, Taina M, Rautiainen S, Könönen M (2015). Diffusion-Weighted Imaging in 3.0 Tesla Breast MRI: Diagnostic Performance and Tumor Characterization Using Small Subregions vs. Whole Tumor Regions of Interest. PLoS One.

[38] Baltzer P, Mann RM, Iima M, Sigmund EE, Clauser P, Gilbert FJ (2020). Diffusion-weighted imaging of the breast - a consensus and mission statement from the EUSOBI International Breast Diffusion-Weighted Imaging working group On behalf of the EUSOBI international Breast Diffusion-Weighted Imaging working group. Eur Radiol.

[39] Yang P, Xu C, Hu X, Shen Y, Hu D, Kamel I (2020). Reduced Field-of-View Diffusion-Weighted Imaging in Histological Characterization of Rectal Cancer: Impact of Different Region-of-Interest Positioning Protocols on Apparent Diffusion Coefficient Measurements. Eur J Radiol.

[40] Sala E, Priest AN, Kataoka M, Graves MJ, McLean MA, Joubert I (2010). Apparent diffusion coefficient and vascular signal fraction measurements with magnetic resonance imaging: Feasibility in metastatic ovarian cancer at 3 tesla technical development. Eur Radiol.

[41] Lindgren A, Anttila M, Rautiainen S, Arponen O, Hämäläinen K, Könönen M (2019). Dynamic contrast-enhanced perfusion parameters in ovarian cancer: Good accuracy in identifying high HIF-1α expression. PLoS ONE.

[42] Marino MA, Helbich T, Baltzer P, Pinker-Domenig K (2018). Multiparametric MRI of the breast: A review. J Magn Reson Imaging.

[43] Feng Y, Liu H, Ding Y, Zhang Y, Liao C, Jin Y (2020). Combined dynamic DCE-MRI and diffusion-weighted imaging to evaluate the effect of neoadjuvant chemotherapy in cervical cancer. Tumori.

[44] Napoletano M, Mazzucca D, Prosperi E, Aisa MC, Lupattelli M, Aristei C (2019). Locally advanced rectal cancer: qualitative and quantitative evaluation of diffusion-weighted magnetic resonance imaging in restaging after neoadjuvant chemo-radiotherapy. Abdom Radiol.

[45] Messina C, Bignone R, Bruno A, Bruno A, Bruno F, Calandri M (2020). Diffusion-weighted imaging in oncology: An update. Cancers (Basel).

[46] Ramspott JP, Baert T, MacKintosh ML, Traut A, Ataseven B, Bommert M (2021). Response evaluation after neoadjuvant therapy: evaluation of chemotherapy response score and serological and/or radiological assessment of response in ovarian cancer patients. Arch Gynecol Obstet [Internet].

[47] Wright AA, Bohlke K, Armstrong DK, Bookman MA, Cliby WA, Coleman RL, Dizon DS, Kash JJ, Meyer LA, Moore KN, Olawaiye AB, Oldham J, Salani R, Sparacio D, Tew WP, Vergote IEM (2016). Neoadjuvant Chemotherapy for Newly Diagnosed, Advanced Ovarian Cancer: Society of Gynecologic Oncology and American Society of Clinical Oncology Clinical Practice Guideline. J Clin Oncol.

[48] Stamoulou E, Spanakis C, Manikis GC, Karanasiou G, Grigoriadis G, Foukakis T (2022). Harmonization Strategies in Multicenter MRI-Based Radiomics. J Imaging.

[49] De Stefano N, Battaglini M, Pareto D, Cortese R, Zhang J, Oesingmann N (2022). MAGNIMS recommendations for harmonization of MRI data in MS multicenter studies. NeuroImage Clin.

